# Ducks induce rapid and robust antibody responses than chickens at early time after intravenous infection with H9N2 avian influenza virus

**DOI:** 10.1186/s12985-019-1150-8

**Published:** 2019-04-11

**Authors:** Jianmei Yang, Hongrui Cui, Qiaoyang Teng, Wenjun Ma, Xuesong Li, Binbin Wang, Dawei Yan, Hongjun Chen, Qinfang Liu, Zejun Li

**Affiliations:** 1 Innovation Team for Pathogen Ecology Research on Animal Influenza Virus, and Department of Avian Infectious Disease, Shanghai Veterinary Research Institute, Chinese Academy of Agricultural Sciences, Shanghai, 200241 People’s Republic of China; 2 Shanghai Key Laboratory of Veterinary Biotechnology, Shanghai, 200240 People’s Republic of China; 3 Department of Diagnostic Medicine/Pathobiology, College of Veterinary Medicine, Kansas, State University, Manhattan, Kansas 66506 USA

**Keywords:** Humoral immune response, Chickens, Ducks, H9N2 AIVs, Different routes

## Abstract

**Background:**

Compared with chickens, ducks are normally resistant to avian influenza virus without clinical signs while they habor almost all subtypes of influenza A viruses. To date, however the mechanism for duck anti-influenza has not been completely understood. The H9N2 avian influenza virus (AIV) is the most prevalent subtype of influenza A virus that infects chickens and ducks in China. However, H9N2 AIV replication and the host immune response in these domestic birds has not been systematically investigated.

**Methods:**

In the present study, we compared the kinetics and magnitudes of antibody responses in chickens and ducks after infection with H9N2 AIV by the intranasal route or intravenous route. Furthermore, we determined the viral replication and distribution in chickens and ducks after infection with H9N2 AIV by the intravenous route.

**Results:**

Our results revealed that the antibody response was rapid and robust in ducks than in chickens at early time (2-3dpi) after intravenous infection with H9N2 AIVs, while delayed and lower antibody detected in ducks than in chickens after intranasal infection with H9N2 AIVs. The virus was detected in multiple organs tissues in chickens but not in ducks infected by the intravenous route.

**Conclusions:**

Our results provide the evidence that humoral immune response could play a critical role in duck resistance for influenza, which expands our knowledge on duck anti-influenza characteristics.

## Background

Chickens and ducks are the two most predominant domestic bird species in China, and they are also the most economically important land fowl and waterfowl as sources of meat, eggs, and feathers. H9N2 AIV was first isolated from turkeys in Wisconsin (USA) in 1966 and was later first identified in chickens in Guangdong province, China in 1994 [1, 2]. Subsequently, viruses of the H9N2 subtype have quickly spread to most areas of China. Currently, H9N2 AIVs have become prevalent among the domestic poultry populations in several Asian countries and are considered to be potential candidates for a future pandemic [3, 4]. Additionally, the H9N2 influenza virus has donated six internal genes to the H7N9 and H10N8 AIVs, which have recently infected humans in China [5, 6]. Furthermore, H9N2 AIVs have been reported to infect pigs and humans, resulting in severe and even lethal cases in humans [7–10]. Although there is currently no evidence of human-to-human transmission of H9N2 AIVs, the results of serological surveillance studies found higher anti-H9 antibody positive rates in serum samples collected from poultry workers [10, 11]. Moreover, there is evidence showing that the continual transmission of H9N2 AIVs between chickens and aquatic birds facilitates the generation of reassortant viruses with the potential to infect humans [12]. This emphasizes that the threat of H9N2 AIVs to public health is a growing concern [10, 13].

H9N2 AIVs continue to circulate in chickens despite the implementation of a long-term vaccination program [3]. Moreover, ducks have been reported to be tolerant to H9N2 AIV infection, since infected ducks typically do not exhibit any clinical symptoms; however, ducks are able to shed the virus and transmit it to other species and can be for almost all types of influenza A viruses [4]. In addition, ducks typically also serve as the natural reservoir for HPAIVs and display no clinical signs following infection, whereas chickens are more susceptible to HPAIVs [14, 15]. Studies have shown that many immune-related genes are involved in the anti-influenza responses of ducks, including innate immune, cellular immune, inflammatory and chemokine genes [16–19]. Ducks have been found to mount more active and robust cellular immune responses compared to chickens exposed to H9N2 AIV by the intranasal route [20]. Following infection with HPAIVs, ducks are able to initiate a faster but lower inflammatory cytokine response followed by the activation of major pattern recognition receptors (i.e., *TLR7*, *RIG-I*, and *MDA-5*) and a persistent cellular response, whereas chickens generate excessive but delayed inflammatory cytokine responses followed by inadequate cellular immune responses, which may result in a higher pathogenicity of the virus in chickens [16]. Other studies have shown that ducks can initiate an immediate and robust response to the HPAIV, A/Vietnam/1203/04 (H5N1), whereas they generate a minimal response to the LPAIV, A/mallard/BC/500/05(H5N2) [21]. Chemokine gene expression is also significantly higher in the lung tissues of ducks infected with HPAIV, compared to those infected with an LPAIV at 1 day post-infection (dpi) [22]. So far, the mechanism for duck anti-influenza is still in dark and has been shown complicated as different results were observed due to different virulent virus strain and viral exposure routes.

Ducks usually have no clinical signs after most LPAI infection, but some H5 HPAIVs have been found to be highly pathogenic (100% lethal) and replicate systemically in ducks [23]. Thus, the current knowledge of AIV infection in ducks should be expanded from respiratory and intestinal infection to systemic infection [23–26]. However, the immune response generated in ducks during systemic infection remains poorly understood. Several studies have evaluated AIV tissue tropism in ducks and turkeys via the intravenous inoculation of AIVs; however, the immune response was not determined following intravenous infection [27].

Since ducks still have some unclear/unknown mechanism for anti-influenza, in the present study, we investigated the antibody responses between chickens and ducks after natural infection and systemic infection using H9N2 AIV model. In addition, the viral titer in the organs was determined in chickens and ducks after infection with H9N2 virus by intravenous route, which was used to mimic a systemic AIV infection in birds. Rapid and robust antibody responses were observed in ducks than in chickens at early time after intravenous infection.

## Methods

### Virus and animals

A/chicken/Shanghai/441/2009 (H9N2), designated SH441, was amplified in nine-day-old specific pathogen-free (SPF) chicken embryos (Beijing Merial Vital Laboratory Animal Technology Co., Ltd., Beijing, China). SH441 was titrated in SPF chicken embryos before it was used for infection or immunization. Allantoic fluids containing the SH441 virus were inactivated with 0.05% β-propiolactone (BPL; Sigma-Aldrich, St. Louis, MO, USA) at 4 °C for 12 h to be used as an inactivated vaccine and was confirmed by infecting in nine-day-old SPF chicken embryos for 3 days to check the HA titer.

Chickens and shelducks aged 10–12 weeks old were hatched from SPF embryos (chicken embryos were obtained from Beijing Merial Vital Laboratory Animal Technology Co., Beijing; duck embryos were obtained from Harbin Veterinary Research, Harbin) and housed in isolators until further analysis.

### Infection of chickens and ducks by intranasal or intravenous route

For each route, two groups were set up, each containing five chickens or five ducks. The same dose was administered to each bird: 1) for the intranasal infection route (mimic viral natural infection), each bird in both groups was intranasally infected with the live SH441 virus at a dose of 10^6^ 50% embryo infectious dose (EID_50_)/100 μL; 2) for the intravenous infection route (viral systemic infection), each bird was intravenously inoculated with the live SH441 virus at a dose of 10^6^ EID_50_/100 μL.

To determine the virus replication in the organs of infected chickens and ducks through the intravenous route, nine chickens and nine ducks were intravenously inoculated with the live SH441 virus at a dose of 10^6^ EID_50_/100 μL. Three birds were necropsied at 1, 2, and 3 dpi, and 13 different tissues (tonsil, spleen, trachea, lung, intestine, brain, thymus, heart, liver, kidney, fabricius, pancreas, and ovary/testis) were collected for further analysis.

### Antibody responses in chickens and ducks exposed to the H9N2 virus

Blood was collected at different time points for each experiment. The serum samples were then used to test the antibody response using both a hemagglutination inhibition (HI) assay, which is the gold standard influenza antibody test, and a blocking ELISA (enzyme linked immunosorbent assay), which we developed previously [28].

The HI assay was performed according to recommendations of the OIE manual. Briefly, the sera were serially diluted (two-fold) in V-bottom 96-well plates and mixed with a standard amount of virus (8 HA units). Then, 0.5% chicken erythrocytes were added to each well and the plates were incubated for 30 min at room temperature. The HI endpoint was the highest serum dilution in which no agglutination was observed.

The blocking ELISA method has been shown to have a high correlation value of 96.9% with the HI assay and had a greater sensitivity for low antibody responses, with concrete numerical values compared to the HI assay [28] Briefly, non-immune sera from SPF chickens and ducks were used as the negative reference sera. Positive reference sera for the H9N2 virus were prepared and stored in our laboratory. The reduction in the OD_450_ value caused by serum antibodies blocking mAb binding was calculated for each sample using the following formula: PI (Percentage of Inhibition, %) = (OD_450_ value of negative reference serum - OD_450_ value of tested serum)/(OD_450_ value of negative reference serum - OD_450_ value of positive reference serum) × 100%.

### Virus titer in the organs of birds following the intravenous infection route

To evaluate viral replication and tissue tropism in chickens and ducks following the intravenous infection, 13 different organ tissues were collected from three chickens and three ducks at 1, 2, and 3 days post-infection (dpi), respectively. Each tissue was weighed and homogenized in PBS containing 100 U/mL penicillin and 100 μg/mL streptomycin at a 1:1 (mL/g) ratio to tissue homogenates, which were centrifuged at 5000 × *g* for 10 min at 4 °C. The supernatants were collected for viral titration in SPF eggs.

We next intra-allantoically injected 9–11-day-old chicken embryonated eggs with 100 μL of the supernatants of tissue homogenates. The viral titer for each organ was determined by the Reed and Muench method and expressed as log_10_ EID_50_/g of tissue [29].

### Statistical analysis

Antibody responses based on HI and blocking ELISA were analyzed by analysis of variance (ANOVA) in GraphPad Prism version 5.0 (GraphPad software Inc., CA,USA). A value of *p* ≤ 0.05 was considered to be significant. Pair-wise mean comparisons between chicken and duck groups were made using Student’s *T* test.

## Results

### Antibody response in birds intranasally infected with the H9N2 virus

**Fig. 1:**
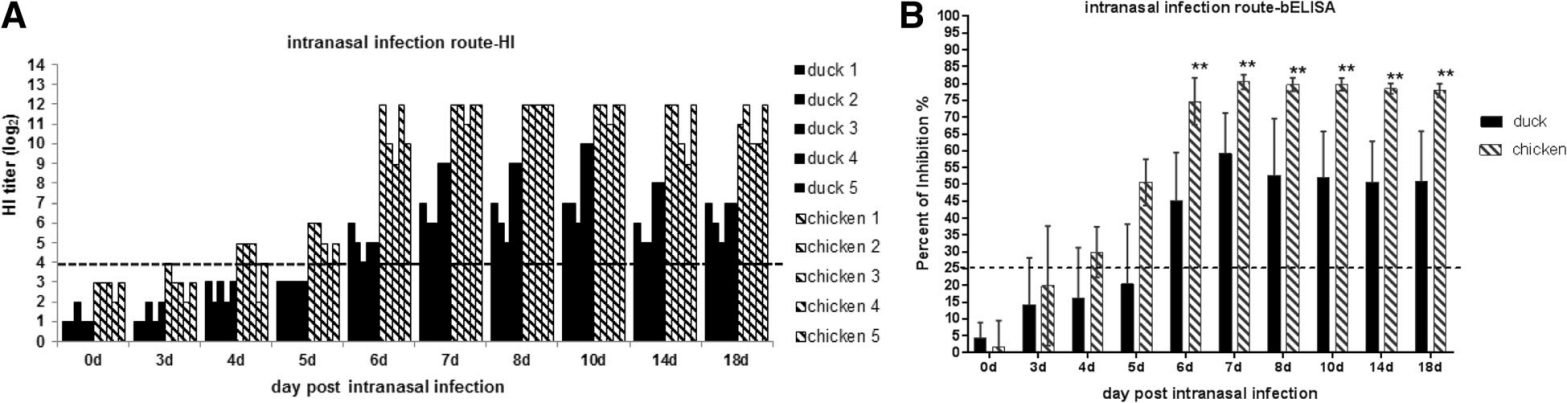
Antibody response in chickens and ducks intranasally infected with the H9N2 virus. **a** A hemagglutination inhibition (HI) assay was performed to test the serum samples of birds at the indicated time points (HI > log_2_4 was considered positive). **b** A blocking ELISA was used to test the serum samples of birds at the indicated time points (PI > 25% was considered positive; **, *p* < 0.01)

Following intranasal infection with the H9N2 virus, three out of five chickens seroconverted at 4 dpi to a positive HI titer (HI > log_2_4) and all chickens (5/5) seroconverted at 6 dpi with a higher HI titer and inhibition according to the results of the blocking ELISA (PI > 25%) (Fig. 1). In contrast, none of the infected ducks seroconverted at 4 dpi and 5 dpi, until four out of five ducks (4/5) were sera positive at 6 dpi and all ducks (5/5) seroconverted at 7 dpi. Noticeably, a significantly higher antibody titer was detected in the chickens than in the ducks from 7 dpi to the experimental end point (18 dpi) (*p* < 0.01) (Fig. 1). These results reveal that ducks display delayed seroconversion (at least 2 days delayed) compared to chickens when they are intranasally infected with the H9N2 virus.

### Antibody response in birds intravenously infected with the H9N2 virus

**Fig. 2:**
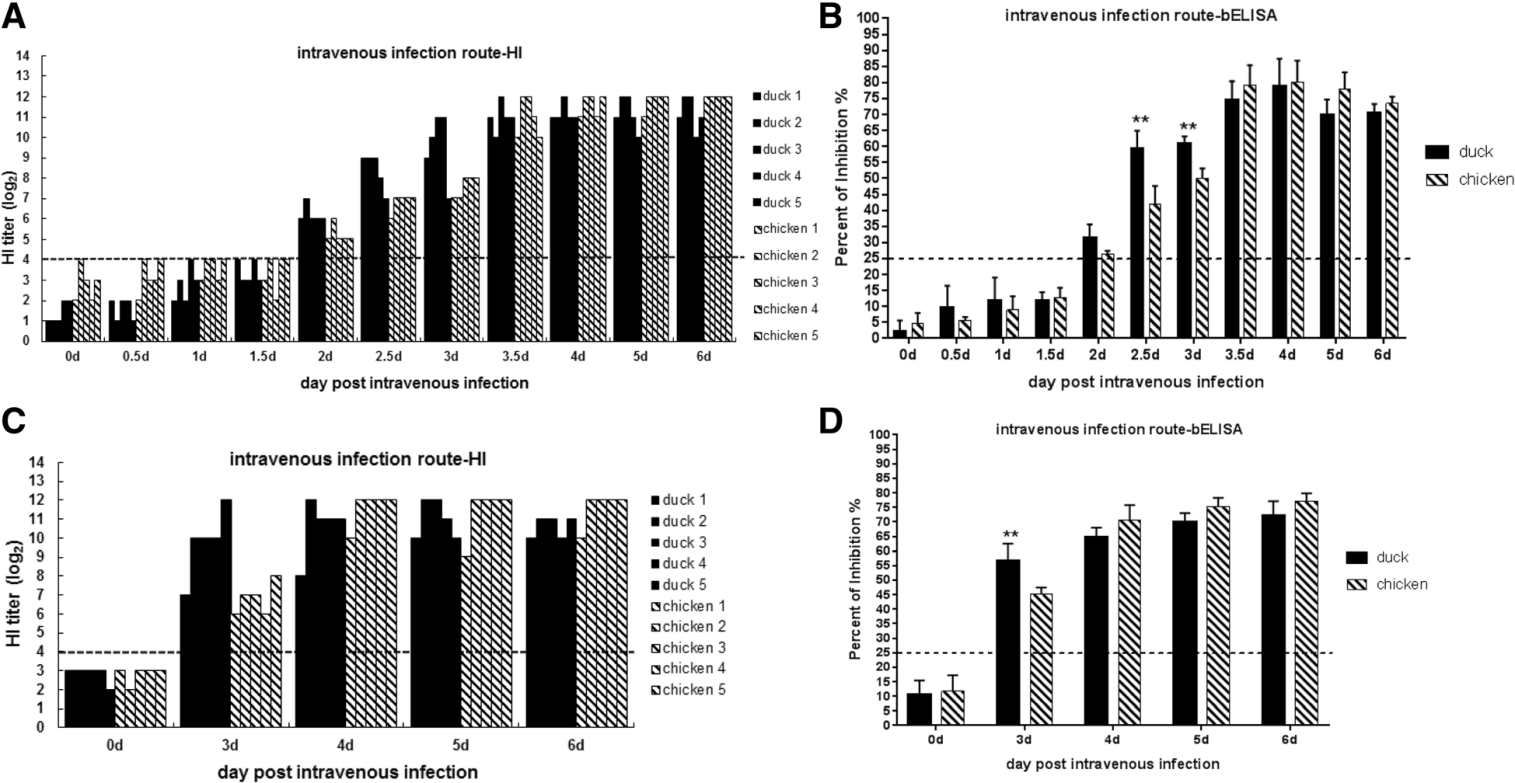
Antibody response in chickens and ducks intravenously infected with inactivated H9N2 virus. **a** and **c** An HI assay was used to test the serum samples of the birds at different time points post-infection as indicated. **b** and **d** A blocking ELISA was used to test the serum samples of birds at different time points post-infection as indicated;**, *p* < 0.01. **a** and **b** present the samples collected at the indicated time points in the same infection experiment. **c** and **d** represent the samples collected at different time points post-infection as indicated in another duplicate independent infection experiment

Following intravenous infection with the H9N2 virus, the antibody response in both ducks and chickens was higher compared to that generated following intranasal infection (Figs. 1 and 2). All of the chickens (5/5) and ducks (5/5) seroconverted at 2 dpi. Noticeably, the antibody titers in the ducks were higher than those in the chickens at the early time points (2 dpi, 2.5 dpi, and 3 dpi). All serum samples were also assessed via a blocking ELISA. Similar to the HI results, the ducks were associated with a significantly higher antibody response than chickens at the early time points (2–3 dpi) following intravenous infection (*p* < 0.01) (Fig. 2a and b). In contrast, the antibody titers in the chickens increased to the same level or moderately higher levels than those exhibited by the ducks from 3.5 dpi to the experimental end point (6 dpi) (Fig. 2a and b). To confirm this finding, another independent experiment was performed by intravenously infecting both types of birds with the H9N2 virus. The antibody titers in the ducks were significantly higher than those in the chickens at 3 dpi (*p* < 0.01), and the antibody titers in chickens increased a little higher than ducks from 4-6dpi (Fig. 2c and d).

### Virus replication in the organs of birds intravenously infected with the H9N2 virus

To our knowledge, this is the first study to show that ducks can produce a significantly higher specific antibody response than chickens following H9N2 virus infection, which was observed by the intravenous infection route (Fig. 2). To further understand how the virus replicates in the infected birds following intravenous infection with H9N2, viral titers were determined by titration in SPF eggs using different tissues collected from infected birds at 1, 2, and 3 dpi.

**Fig. 3:**
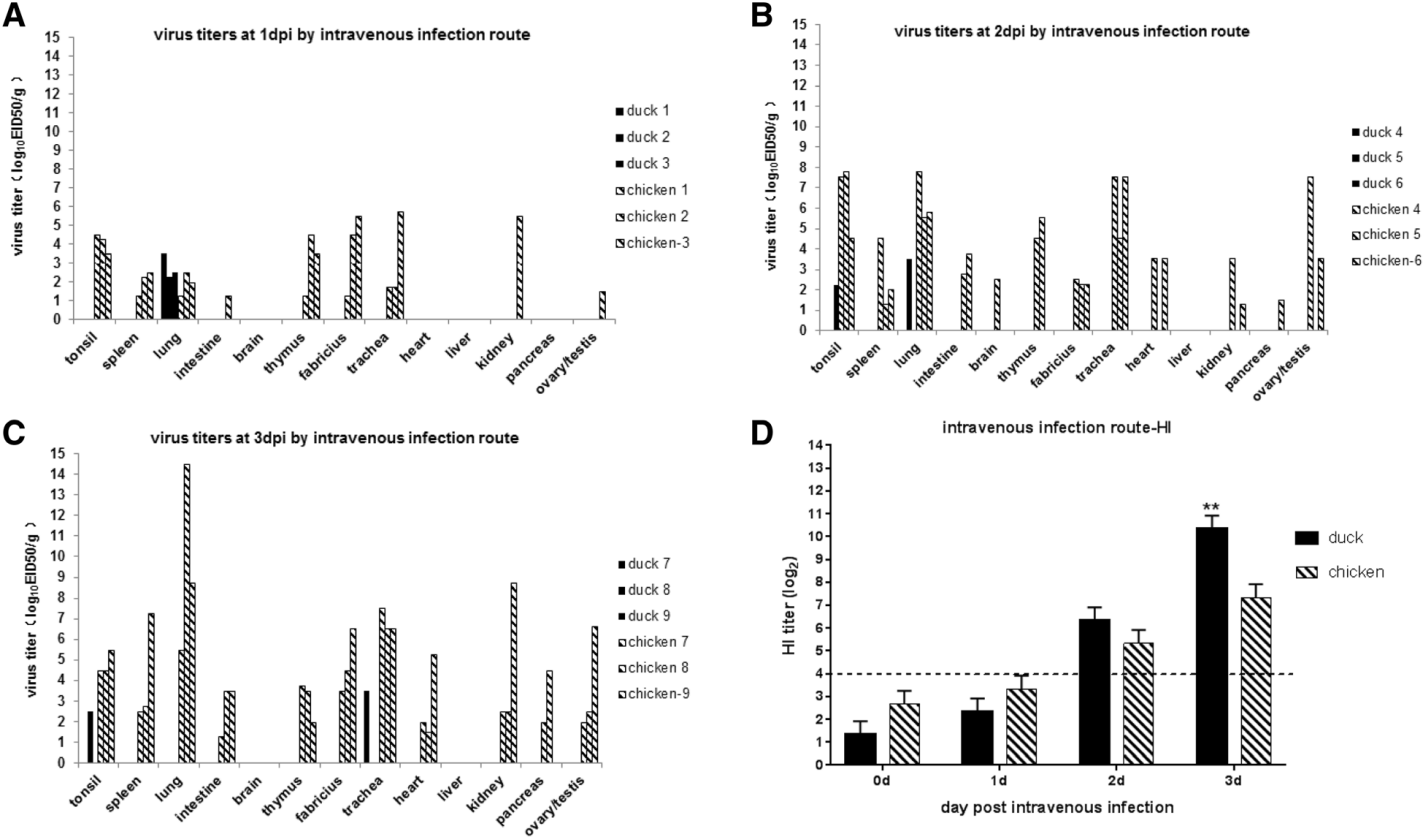
Virus titer and HI antibody responses of chickens and ducks intravenously infected with the H9N2 virus. **a** - **c** Virus titers were determined in 13 different organs of birds at the indicated time points: 1 dpi (**a**), 2 dpi (**b**), and 3 dpi (**c**). **d** An HI assay was used to test the serum samples of the birds at the indicated time points. dpi: days post-infection

After intravenous infection with the H9N2 virus, the chickens exhibited a systemic infection characterized by higher viral replication titers and a wider tissue tropism compared to the ducks. At 1 dpi, the virus was detected in the tonsil, spleen, thymus, fabricius, and trachea of all (3/3), as well as in one intestine, kidney, and testis (1/3) sample collected from the chickens (Fig. 3a - c & Table 1). At 2 dpi, the virus was also detected in the brain (1/3), heart (2/3), and pancreas (1/3) samples from the chickens, and viral titers were substantially increased at 3 dpi in all of these tissue samples (Fig. 3b-c & Table 1). In contrast, at 1 dpi, the virus was detected in only three duck lungs (3/3), with no virus detected in any of the other duck organs. At 2 dpi, the virus was only detected in one out of three duck lungs (1/3) and tonsil (1/3) samples. At 3 dpi, the virus was detected in one out of three duck tonsil (1/3) and trachea (1/3) samples, and no virus replication was detected in the duck lungs at 3 dpi (0/3) (Fig. 3a-c & Table 1). The viral titers were substantially higher in all chicken organs compared with the duck organs, except for the duck lungs at 1 dpi. Viral titers were higher in the chicken lungs and tracheas at 2 dpi and 3 dpi with 4.5–14.5 log_10_ EID_50_/g tissue (Fig. 3b-c).

**Table 1:** Virus titers determined by titration in SPF eggs for different tissues from chickens and ducks intravenously infection with H9N2 virus

Time	Animal	Virus titers in different tissues (log_10_EID_50_/g)												
		tonsil	spleen	lung	intestine	brain	thymus	fabricius	trachea	heart	liver	kidney	pancreas	Ovary (testis^b^)
1 dpi	Duck 1	/^a^	/	3.5	/	/	/	/	/	/	/	/	/	/
	Duck 2	/	/	2.25	/	/	/	/	/	/	/	/	/	/
	Duck 3	/	/	2.5	/	/	/	/	/	/	/	/	/	/
	Chicken 1	4.5	1.25	1.25	/	/	1.25	1.25	1.75	/	/	/	/	/^b^
	Chicken 2	4.25	2.25	2.5	1.25	/	4.5	4.5	1.75	/	/	5.5		1.5^b^
	Chicken 3	3.5	2.5	1.98	/	/	3.5	5.5	5.75	/	/	/	/	/
2 dpi	Duck 4	/	/		/	/	/	/	/	/	/	/	/	/
	Duck 5	/	/	3.5	/	/	/	/	/	/	/	/	/	/
	Duck 6	2.25	/	/	/	/	/	/	/	/	/	/	/	/
	Chicken 4	7.5	4.5	7.75	/	2.5	4.5	2.5	7.5	3.5		3.5		7.5
	Chicken 5	7.75	1.25	5.5	2.75	/	5.5	2.25	4.5	/	/	/	/	/
	Chicken 6	4.5	1.98	5.75	3.75			2.25	7.5	3.5		1.25	1.5	3.5
3 dpi	Duck 7	/	/	/	/	/	/	/	3.5		/	/	/	/
	Duck 8	2.5	/	/	/	/	/	/	/	/	/	/	/	/
	Duck 9	/	/	/	/	/	/	/	/	/	/	/	/	/
	Chicken 7	4.5	2.5	5.5	1.25	/	3.75	3.5	7.5	1.98	/	2.5	1.98	1.98^b^
	Chicken 8	4.5	2.75	14.5	3.5	/	3.5	4.5	6.5	1.5	/	2.5	4.5	2.5^b^
	Chicken 9	5.5	7.25	8.75	3.5	/	1.98	6.5	6.5	5.25	/	8.75	/	6.64^b^

^a^Note: /, means no virus was detected from this sample in SPF eggs; and other numbers in this table are described as log_10_EID_50_/g for their virus titers

^b^, means this sample was from duck or chicken testis

In addition, the antibody responses were also determined by an HI assay. The ducks produced higher antibody titers than the chickens at 2 dpi and 3 dpi, especially at 3 dpi (*p* < 0.01), which was consistent with the findings as we previously described in this study (Figs. 2 and 3d).

## Discussion

In contrast to chickens, ducks are normally resistant to most influenza A virus infections and do not exhibit clinical symptoms; however, the mechanisms associated with duck anti-influenza remain poorly understood. Based on the current available knowledge, both local mucosal and innate immunity are considered to play a critical role in duck anti-influenza responses [17, 21, 30]. Although the innate immune response is highly effective for antiviral infection, it is not specific or sufficient for providing a defense against viral infection, that is why ducks can be for almost all subtypes of influenza A viruses. The acquired immune response activated by MHC molecules is critical for eliminating viral infections through the generation of virus-specific antibodies. However, the specific antibody responses of chickens and ducks infected with AIVs have not been fully characterized. Thus, we aimed to investigate such responses in this study.

Some researchers showed that ducks had a deficit in adaptive antibody to AIV because of genetics-linked characteristics, which may contribute to weak antibody responses and the perpetuation of the virus in this animal reservoir [31]. In this present study, ducks really showed the weaker antibody responses including delayed seroconversion and lower HI antibody titers than chickens by intranasal infection route (viral natural infection),which is consistent with previous findings. However, by intravenous infection route (viral systemic infection), ducks inversely could produce robust antibody responses than chickens with antibody levels at early time after infection (2, 2.5, 3dpi). In previous studies, ducks could also show strong and sensitive antibody responses when exposed to attenuated viruses [32–34]. The specific antibody response could be detected as early as at 3-6 dpi in ducks by intranasal or intramuscular immunization with DTMU (duck Tembusu) attenuated viruses [32]. A marked increased level of DEV-specific IgG antibodies in duck serum were detected as early as at 6dpi by orally infection with virulent DEV (duck enteritis virus) Chv strain [34] or byvaccinated with DEV Cha strain [33]. These results indicated that ducks actually could have strong ability in their antibody response when exposed to virus infection or vaccination by some routes, which were also evidenced by our findings of ducks could produce higher antibody response than chickens at early time than chickens by intravenous infection of H9N2 AIVs (Figs. 2 and 3).

In this study, ducks displayed delayed seroconversion with a lower HI antibody titer compared with chickens infected with the same dose of virus via the intranasal route (viral natural infection). In order to mimic the systemic infection in chickens and ducks, we infected chickens and ducks through the intravenous route. The results showed that live H9N2 viruses induced a faster and higher antibody response compared to the intranasal route (Figs.1 and 2). Interestingly, the ducks exhibited an unexpectedly higher antibody titer compared to chickens at the earlier time points (2–3 dpi) when they were infected intravenously. However, a similar antibody response was observed in both species at the later time points, which was confirmed by independent and different assays (Figs. 2 and 3).

Furthermore, following intravenous infection, chickens but not ducks displayed a systemic infection, as the virus was detected in 12 out of 13 organs collected in the infected chickens with a higher titer compared to the limited detection in ducks. The lung and trachea were the most severely affected organs in the chickens, followed by the fabricus (bursa), tonsil, and spleen (Fig. 3). The viral tropism was narrow in some duck respiratory and immune organs, as viral replication could only be detected in the lungs (3/3 at 1 dpi and 1/3 at 2 dpi), trachea, (1/3 at 3 dpi), and tonsil (1/3 at 3 dpi) of the ducks. In addition, the viral titers in the ducks were quite low and the virus was quickly cleared from the duck lungs at 3 dpi (0/3 at 3 dpi) (Fig. 3). A previous study isolates of pheasant and turkey origin isolates were more pathogenic in turkeys, but had limited distributions and effects in ducks after intravenous inoculation [27]. Moreover, the pancreas was the most severely affected organ in turkeys, followed by the kidney and liver, whereas the spleen and bursa were the most commonly affected organs in ducks [27], which also suggested that immune organs of ducks were the targets of influenza A virus after intravenous infection as shown in this study (Fig. 3).

Based on our knowledge, this is the first study to reveal that intravenous infection with the LPAIV H9N2 virus can induce a rapid and robust humoral immune response in ducks at early time points (2-3dpi), but not in chickens, in which a systemic infection is observed, similar to HPAIV infection. In contrast no systemic infection was observed in ducks.

The ducks are resistant to most influenza A virus infection, which is related with innate immunity, and most likely also associates with the specific antibody responses as shown in this present study. The robust antibody response at 2-3dpi detected in ducks could be helpful for limiting virus spread in vivo, so the virus replicated poorly in ducks compared with in chickens. However the mechanism of the rapid and robust antibody responses in ducks infected through the intravenous route than in chickens at early time remains unclear and needs to be investigated in the future studies.

## Conclusion

Our results showed that, compared to chickens, following intranasal infection with an H9N2 AIV, the antibody immune response was delayed in ducks, whereas a rapid and robust humoral immune response was induced at early time points in ducks. when intravenously infected with the H9N2 AIV. Furthermore, the virus was detected in multiple organs in chickens but not in ducks infected through the intravenous route. These findings provide the evidence that the humoral immune response could play an important role in duck resistance for influenza, and expand our current knowledge on duck anti-influenza characteristics.

## Abbreviations

AIV: Avian influenza virus
DEV: Duck enteritis virus
dpi: Day post-infection
DPV: Duck plague virus
DTMUV: Duck tembusu virus
EID_50_: 50% embryo infectious dose
ELISA: Enzyme linked immunosorbent assay
HI: Hemagglutination inhibition
HPAIV: Highly pathogenic avian influenza virus
LPAIV: Low pathogenic avian influenza virus
OIE: Office International Des Epizooties
PI: Percentage of inhibition
SPF: Specific pathogen-free
